# Spinal dysraphism and dislocated hip: Beware of anomalous sciatic nerve through Ilium, a case report

**DOI:** 10.1097/MD.0000000000009770

**Published:** 2018-03-23

**Authors:** Amanda T. Whitaker, James Kasser, Young-Jo Kim

**Affiliations:** a Nationwide Children's Hospital, Department of Orthopaedic Surgery, Columbus, OH; b Boston Children's Hospital, Department of Orthopaedic Surgery, Boston, MA.

**Keywords:** fibular hemimelia, hip dysplasia, sciatic nerve, spinal dysraphism

## Abstract

**Rationale:**

The sciatic nerve runs a predictable course combining L4-S3 nerve roots through the true pelvis and under the greater sciatic notch. There are reports of bony protuberances from the sacrum and ilium in cases of spinal dysraphism; however advanced imaging, treatment, or outcomes are not described. There are no cases with associated fibular hemimelia in the current literature.

**Patient concerns:**

This is a 4-year-old girl with tethered cord, acetabular dysplasia with hip subluxation, congenital short femur, anterior cruciate ligament (ACL) deficiency, and fibular hemimelia with her sciatic nerve coursing through the ilium.

**Diagnosis:**

Aberrant course of the sciatic nerve through the ilium in the setting of spinal dysraphism.

**Outcomes:**

The hip subluxation was treated with a femoral varus derotation osteotomy and Salter osteotomy with transposition of the sciatic nerve into the greater sciatic notch resulting in a stable hip with no sciatic nerve symptoms at last follow-up.

**Lessons:**

The combination of spinal dysraphism with acetabular dysplasia should be a warning for anomalous sciatic nerveanatomy, possibly through the ilium. Preoperative imaging (MRI, CT scan) may be obtained and carefully reviewed for the course of the sciatic nerve prior to pelvic or femoral osteotomy. Decompressing the sciatic nerve from the aberrant foramen may be considered as part of the procedure.

## 1 Introduction

The sciatic nerve runs a predictable course from the joining of the ventral rami of L4-S3 nerve roots, through the true pelvis and under the greater sciatic notch, with variability to where it divides into its terminal branches.^[1,2]^ However, the sciatic nerve is reliably found in the greater sciatic notch.^[1,2]^ We describe a case of an aberrant sciatic nerve through the ilium in a child with spinal dysraphism, fibular hemimelia, and hip dysplasia with dislocation. This is the first report in the literature of this sciatic nerve variant with fibular hemimelia and raises awareness of the association of spinal dysraphism and abnormal course of the sciatic nerve when reconstructing a subluxated hip.

## 2 Case

Our patient is a 4-year-old girl who presents with a painful left hip. She has been followed for left acetabular dysplasia and anterior hip subluxation since birth. She had previously been treated with a hip abduction orthosis for hip dysplasia. The parents wanted to wait for surgical intervention, however the pain prompted treatment.

Her medical history is complicated by multiple congenital anomalies of the lumbar spine and tethered spinal cord, which had previously undergone release with complication of postoperative neurogenic bladder. She has left fibular hemimelia and underwent a Syme amputation for her associated foot deformity. Her left leg is also anterior cruciate ligament (ACL) deficient with a congenitally short left femur.

On earliest exam, she held her leg in a flexed and abducted position with a 40° flexion contracture at the hip. Her left femoral head easily subluxated anteriorly, and the femoral head was palpable. She had hypoplastic gluteal musculature that persisted throughout growth. At the age of 4, when actively flexing the hip, she externally rotates the hip maximally. The left hip had good flexion and abduction. She was a household ambulator with a prosthesis on her left leg.

**Figure 1:**
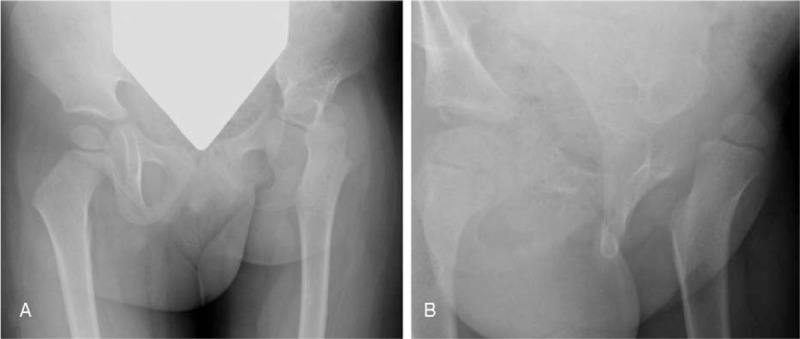
(A) AP pelvis at age 3 with left hip acetabular dysplasia and anterior dislocation of femoral head best seen on oblique view (B) with a small femoral epiphysis. AP = anterior–posterior.

**Figure 2:**
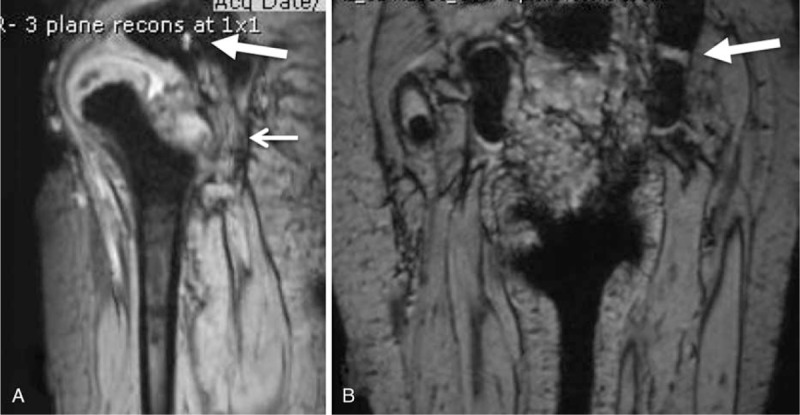
T2-weighted MRI (A) Sagittal MRI with thick arrow pointing to the anomalous foramen and the thin arrow at the course of the sciatic nerve. (B) Coronal MRI with thick arrow at the anomalous foramen containing the sciatic nerve. MRI = magnetic resonance imaging.

On early ultrasound, she had a dysplastic hip with the iliac line, usually straight on ultrasound, demonstrating a curved anatomic variant. Radiographs demonstrate acetabular dysplasia and subluxation of the femoral head (Fig. 1). Due to her anterior subluxation and unusual nature of hip instability, a 3-dimensional (3D) model was created from a high-resolution magnetic resonance (MR) scan using 3D printer to examine the relationship between the lesser trochanter and the proximal femur for the planning of the location of the osteotomy. Her anteversion located between the lesser trochanter and the proximal femur measured 72π with subluxation in the anterior and superolateral direction. The left hemipelvis was hypoplastic with an abnormal cleft transversing the left iliac bone just superior to the acetabulum. She had an absence of the left gluteal muscles (Fig. 2). The left quadriceps and hamstrings noted to be atrophied with less atrophy of the adductors, psoas, and iliacus. The left sciatic nerve was not able to be identified by the radiologist preoperatively.

**Figure 3:**
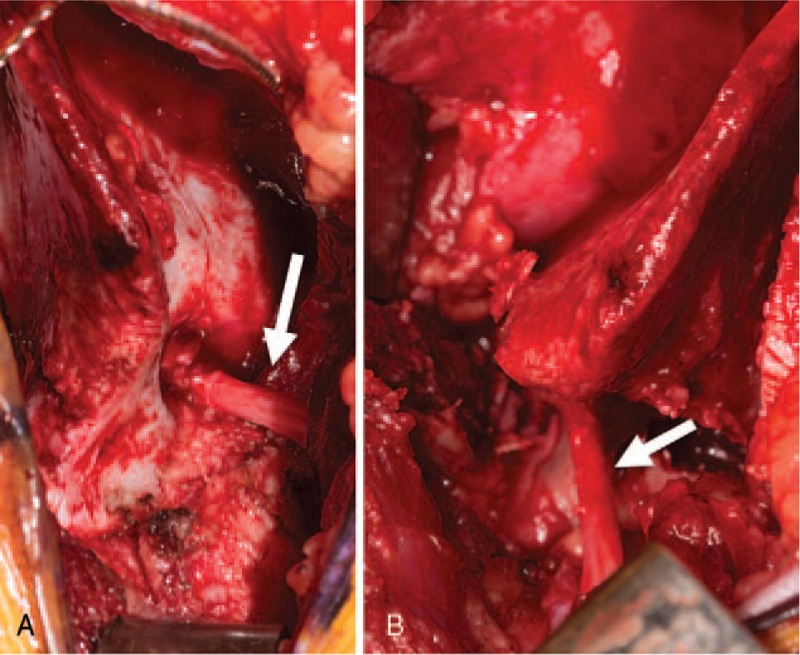
Intraoperative photographs of the sciatic nerve with (A) demonstrating the course of the nerve (arrow) through the anomalous foramen though the ilium and (B) after decompression and iliac osteotomy.

**Figure 4:**
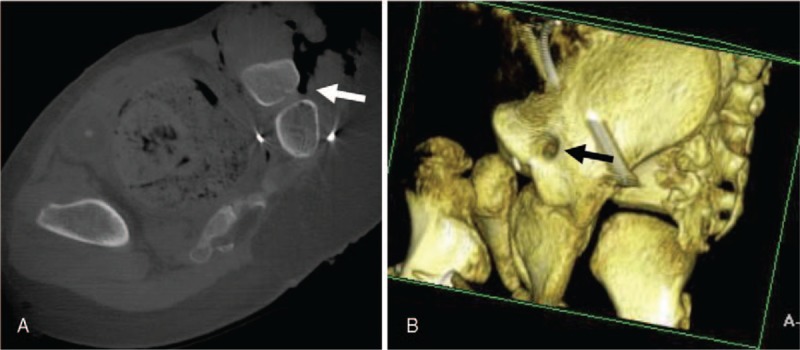
Intraoperative CT scan. (A) Axial cuts demonstrating anomalous foramen and (B) 3D reconstruction illustrating the anatomy of the ilium with foramen. CT = computer tomography.

**Figure 5:**
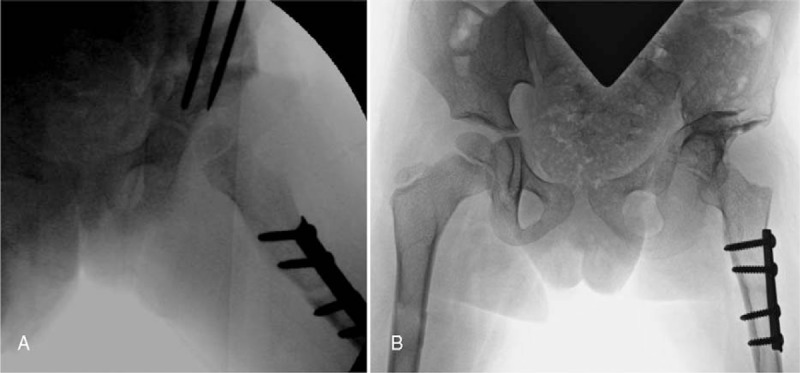
Postoperative (A) fluoroscopy frog-lateral demonstrating reduction of the femoral head in the acetabulum with good acetabular coverage and (B) 14 months postoperative AP pelvis after removal of pelvic hardware with good maintenance of reduction of the femoral head and acetabular coverage.

To correct her hip subluxation and acetabular dysplasia, the proximal femoral derotational osteotomy of 40° was completed. During the iliac dissection for the innominate osteotomy, a nerve was identified exiting through a foramen in the ilium (Fig. 3A). Upon stimulation, the nerve was identified as the sciatic nerve. An intraoperative low-dose CT scan was completed to confirm the location of the foramen and further plan the iliac osteotomy (Fig. 4). The iliac osteotomy was made to the foramen in the ilium containing the sciatic nerve. The sciatic nerve was mobilized. The osteotomy was then completed inferior to the nerve using a Kerrison ronguer. The osteotomy was mobilized, and the sciatic nerve was placed in the sciatic notch after correcting the acetabulum (Fig. 3B). The hip was stable after fixation with 2 threaded Kirschner wires (Fig. 5A).

Postoperatively, she had hypersensitivity at the distal end of her residual limb, which resolved after 6 weeks. She is doing well and ambulating as was preoperatively at 14 months (Fig. 5B). Ethical approval was not necessary due to the nature of this retrospective case report.

## 3 Discussion

Iliac anomalies decreasing the size of the greater sciatic notch in spinal dysraphism have been reported in radiology literature four decades ago.^[3–6]^ There are 2 cases reported of the sciatic nerve through the ilium with compression, but may be the same patient.^[3,4]^ The nerve was decompressed by removing the inferior portion of the bone to expand the sciatic notch in contrast to our Salter pelvic osteotomy.^[4]^ Nine cases have been described with associated hip subluxation and acetabular dysplasia; however, no treatment options or intraoperative findings have been described.^[3–9]^ Most often, the iliac anomalies increase bony formation on the sacrum and/or ilium, often seen as bony protuberances.^[3–9]^ Our case is the first to treat the acetabular dysplasia and hip dislocation with transposition of the sciatic nerve into the greater sciatic notch. Also, this is the first described case of spinal dysraphism, anomalous sciatic nerve, and fibular hemimelia.

The combination of spinal dysraphism with acetabular dysplasia should be a warning for anomalous sciatic nerve anatomy, possibly through the ilium. Preoperative imaging (MRI, CT scan) may be considered and carefully reviewed for the course of the sciatic nerve with attention to the ilium prior to pelvic or femoral osteotomy. Consider decompressing the sciatic nerve from the aberrant foramen as part of the procedure.

## 4 Author contributions

**Conceptualization:** A.T. Whitaker, J. Kasser, Y-J. Kim.

**Data curation:** A.T. Whitaker, Y-J. Kim.

**Formal analysis:** A.T. Whitaker, Y-J. Kim.

**Investigation:** A.T. Whitaker, Y-J. Kim.

**Methodology:** A.T. Whitaker.

**Resources:** A.T. Whitaker, J. Kasser, Y-J. Kim.

**Software:** Y-J. Kim.

**Supervision:** J. Kasser, Y-J. Kim.

**Writing – original draft:** A.T. Whitaker.

**Writing – review & editing:** A.T. Whitaker, J. Kasser, Y-J. Kim.
